# NR3C1 Modulates Wnt Signalling to Influence the Invasiveness and Immune Features of Nonfunctioning Invasive Pituitary Adenomas

**DOI:** 10.1111/jcmm.71097

**Published:** 2026-03-29

**Authors:** Xiaoping Wang, Jinfeng Zhang, Tao Jiang, Zhijun Yang, Yu Zhang, Pinan Liu, Yuanxiang Lin

**Affiliations:** ^1^ Department of Neurosurgery The First Affiliated Hospital of Fujian Medical University Fuzhou City Fujian Province China; ^2^ Department of Neurosurgery Xiamen Hospital of Traditional Chinese Medicine Xiamen Fujian Province China; ^3^ Department of Neurosurgery Beijing Tiantan Hospital, Capital Medical University Beijing China

**Keywords:** diagnostic biomarker, immunity, nonfunctioning invasive pituitary adenomas, NR3C1

## Abstract

Pituitary adenomas (PAs) are common intracranial tumours, and invasiveness in nonfunctioning invasive pituitary adenomas (NIPAs) predicts poor prognosis. The molecular mechanisms driving this phenotype remain unclear. This study explored the role of nuclear receptor subfamily 3 group C member 1 (NR3C1) in NIPA invasiveness and its regulation of Wnt signalling. mRNA expression profiles of 32 PA samples were generated by RNA‐seq, and proteomic data from 19 samples were obtained by mass spectrometry. Immune‐related differentially expressed genes (DEGs) were retrieved from GeneCards. Weighted gene coexpression network analysis identified modules and hub genes linked to invasiveness, while machine learning methods (support vector machine, LASSO, random forest) prioritised key genes. Gene set enrichment analysis (GSEA) assessed pathways associated with candidate gene expression. NR3C1 expression and function were validated by immunohistochemistry, Western blotting and invasion assays. Integration of transcriptomic, proteomic and immune‐related datasets yielded 11 overlapping genes, with NR3C1 emerging as the top candidate. NR3C1 was significantly upregulated in NIPAs and demonstrated good discriminatory power by ROC analysis. GSEA associated high NR3C1 expression with Wnt pathway activation. Functional experiments confirmed that NR3C1 overexpression enhances the invasive capacity of PA cells. NR3C1 promotes the invasive phenotype of NIPAs by activating Wnt signalling. These findings suggest NR3C1 as a potential biomarker and therapeutic target for invasive pituitary adenomas.

## 1 Introduction

Pituitary adenomas (PAs) are common intracranial tumours, accounting for approximately 15% of all such tumours [1]. They are categorised as functional or non‐functional adenomas based on hormone secretion and biological behaviour. Functional adenomas include growth hormone‐secreting adenomas, adrenocorticotropic hormone‐secreting adenomas and prolactinomas, which cause excessive hormone release and associated clinical manifestations [2]. In contrast, non‐functional adenomas generally do not secrete hormones and are often incidentally discovered as mass lesions on imaging studies. They represent about 30% to 50% of PAs [3]. Patients with NIPAs commonly present with visual disturbances, headaches and hypopituitarism, reflecting the tumour's invasive nature [4, 5]. A study involving 146 NIPA patients showed that tumour size in NIPAs was significantly larger than in non‐functional non‐invasive pituitary adenomas (NFPAs), and these tumours were more likely to cause visual impairment and other neurological deficits [6]. Although previous studies suggest that NIPA invasiveness is linked to multiple molecular mechanisms, the precise underlying pathways remain insufficiently understood, particularly at the molecular level [7]. Therefore, elucidating the invasive mechanisms of NIPAs has substantial clinical significance.

The immune microenvironment plays a pivotal role in the development and progression of PAs. Tumour‐infiltrating immune cells (TIICs), including macrophages and T cells, can profoundly influence tumour behaviour and patient prognosis [8, 9]. The composition and function of TIICs vary significantly across different pituitary adenoma subtypes, correlating closely with tumour invasiveness and recurrence rates [10, 11]. For example, CD68^+^ macrophage infiltration positively correlates with tumour size and invasiveness, while CD8^+^ T cell density is linked to prognosis [12]. High CD8^+^ T cell infiltration generally predicts better outcomes, whereas elevated regulatory T cell (Treg) levels are associated with poor prognosis, highlighting the importance of immune balance in tumour progression [13]. Accordingly, modulating the immune microenvironment may offer a novel therapeutic avenue to enhance anti‐tumour responses.

Nuclear receptor subfamily 3 group C member 1 (NR3C1), commonly known as the glucocorticoid receptor (GR), is a ligand‐activated transcription factor mediating glucocorticoid effects via downstream signalling regulation [14]. Its regulatory mechanisms within the tumour microenvironment are complex. For instance, psychological stress can promote ovarian cancer metastasis via NR3C1 activation involving its interaction with nuclear protein 1 (NUPR1) [15]. In colorectal cancer, NR3C1 expression is regulated by DNA methylation, with low NR3C1 levels associated with increased tumour proliferation and metastasis [16, 17]. However, the role of NR3C1 in NIPAs remains unexplored.

Current research on NIPAs largely centres on genomic and transcriptomic analyses. Although the Wnt signalling pathway is implicated in various tumours, its involvement in NIPAs is unclear [18]. NR3C1, as a regulatory factor, may interact with the Wnt pathway, providing a novel perspective on NIPA invasiveness [19]. Multi‐omics analysis investigating NR3C1 and its regulation of Wnt signalling could identify new biomarkers and therapeutic targets for NIPA diagnosis and treatment.

In this study, we employed RNA sequencing, mass spectrometry, weighted gene co‐expression network analysis (WGCNA) and machine learning to comprehensively analyse NIPA samples and construct gene and protein expression profiles. Clinical data were used to validate findings and elucidate NR3C1 function and regulatory mechanisms in NIPAs. The study aims to clarify NR3C1's impact on NIPA invasiveness and its regulation of the Wnt signalling pathway, offering potential biomarkers and therapeutic targets. Functional experiments using the NR3C1/GR agonist dexamethasone [20] and the selective NR3C1/GR antagonist RU486 [21] further validated NR3C1/GR's specific role in NIPA progression. By elucidating NR3C1's role and signalling complexity in NIPAs, this work seeks to inform more effective clinical treatment strategies and improve patient prognosis.

## 2 Materials and Methods

### 2.1 Data Sources

The mRNA libraries used in this study were constructed from postoperative tissue samples of 15 patients with NIPAs and 17 patients with NFPAs. Detailed experimental procedures can be found at the BGI experimental protocol link (https://www.yuque.com/yangyulan‐ayaeq/oupzan/gu8ls7). Ultimately, we obtained mRNA expression profiles for 32 pituitary adenoma samples, which were uploaded to the Gene Expression Omnibus (GEO) database (accession number GSE260487). Proteomics data were derived from postoperative tissue samples obtained from 19 patients diagnosed with pituitary adenoma, including 10 NIPAs and nine NFPAs. By preparing protein supernatants from these tissue samples and analysing them using liquid chromatography–tandem mass spectrometry (LC–MS/MS), we obtained quantitative proteomics data for all pituitary adenoma samples. These data have been uploaded to the ProteomeXchange database (accession number PXD039328). We retrieved 1736 immune‐related genes from the GeneCards database (see Table S1). The Limma package (version 3.62.1; https://www.bioconductor.org/packages/release/bioc/html/limma.html) was used to screen for differentially expressed genes (DEGs) between NIPAs and NFPAs groups, with a false discovery rate (FDR) < 0.05 and |log2 fold change (FC)| ≥ 1 set as the screening criteria. For the proteomic analysis, MaxQuant software was used, with screening criteria set at |log2 fold change| > 1 and a *p*‐value < 0.05. Volcano plots were generated using the ggplot2 package (version 3.5.1; https://cran.r‐project.org/web/packages/ggplot2/index.html). An external validation gene set, GSE169498, which includes tissue samples from 49 patients with NIPAs and 24 patients with NFPAs, was used for further validation of our findings.

### 2.2 Weighted Gene Co‐Expression Network Analysis (WGCNA)

WGCNA is a method widely used in systems biology to construct co‐expression networks by correlating changes in gene expression signals with clinical phenotypes. In our study, we used the WGCNA package (version 1.73; https://cran.r‐project.org/web/packages/WGCNA/index.html) to construct gene co‐expression networks. To ensure network reliability, we filtered the top 25% of genes with the highest variance. Pearson correlation coefficients between genes were calculated, and a soft‐thresholding power β was chosen to achieve a scale‐free network fit index close to 0.9, enhancing the conformity of the network construction to scale‐free topology standards. The adjacency matrix was transformed into a topological overlap matrix (TOM). Hierarchical clustering techniques were used to generate a hierarchical clustering tree of genes. To obtain stable module detection results, the minimum module size (minModuleSize) was set to 50. Modules were identified using the dynamic tree cut method, and similar modules were merged based on module eigengene correlations. After module identification, we calculated the gene significance (GS) and module membership (MM) for each gene. To identify modules closely related to clinical features, we calculated the correlation between modules and clinical traits and visualised the results. Focus was placed on modules and genes significantly associated with pituitary adenoma invasiveness.

### 2.3 Machine Learning

To prioritise the identification of genes associated with pituitary adenoma invasiveness, we employed three machine learning algorithms: Support Vector Machine‐Recursive Feature Elimination (SVM‐RFE), LASSO logistic regression analysis and the random forest (RF) algorithm to screen for feature genes. We used the SVM‐RFE method, an embedded method based on support vector machines, which identifies optimal variables by eliminating features from the SVM‐generated feature vector. To improve learning performance and allow for further gene screening, we utilised the SVM‐RFE method from the e1071 package (version 1.7–16; https://cran.r‐project.org/web/packages/e1071/index.html). We applied LASSO logistic regression analysis, which identifies variables by finding the *λ* value that minimises the classification error. The glmnet package (version 4.1‐8) was used for LASSO computation. LASSO analysis constructs generalised linear models and aids in variable selection, thereby identifying the characteristic variables for the optimal classification model. We used the RF algorithm to calculate the mean decrease Gini importance for each feature. RF operates by ensemble learning of multiple decision trees and was used to rank the features; a value above 0.25 was considered indicative of typical importance above chance. Through the intersection of results from these three machine learning methods, we identified the screened feature genes, which are considered core genes closely associated with pituitary adenoma invasiveness.

### 2.4 Immune‐Related Research

To explore the impact of our target gene on tumour immunity, we conducted an assessment of immune cell composition across different samples by e1071 package (version: 1.7–16, https://cran.r‐project.org/web/packages/e1071/index.html), preprocessCore package (version: 1.68.0, https://bioconductor.org/packages/release/bioc/html/preprocessCore.html) and CIBERSORT algorithm. This analysis was visually represented using bar charts to highlight any variations. Additionally, we employed the CIBERSORT algorithm to examine the relationship between the target gene and diverse immune infiltrating cells within the tumour microenvironment. The results were visualised graphically using the corrplot (version: 0.95, https://cran.r‐project.org/web/packages/corrplot/index.html) and the vioplot (version: 0.5.0, https://cran.r‐project.org/web/packages/vioplot/index.html) packages. Furthermore, utilising software packages like limma, we investigated the association between our target genes and the expression of immune checkpoint molecules, where common immune checkpoint‐related genes were obtained from the literature.

### 2.5 Cell Culture and Cell Transfection

The pituitary adenoma cell line HP75 was purchased from the American Type Culture Collection (ATCC, Manassas, VA, USA). Cells were cultured in Dulbecco's Modified Eagle Medium/Ham's F12 nutrient mixture (Thermo Fisher Scientific, USA) supplemented with 10% fetal bovine serum, and confirmed to be free of bacterial and mycoplasma contamination as per relevant instructions. Cells were maintained in a humidified incubator with 5% CO_2_. For transfection, three siRNAs targeting the NR3C1 transcript were designed and synthesised. The specific sequences for NR3C1 siRNA were as follows: NR3C1 siRNA #1 (5′‐GAAGTGTTATATGCAGGATAT‐3′), NR3C1 siRNA #2 (5′‐GAAGTGTTATATGCAGGATAT‐3′) and NR3C1 siRNA #3 (5′‐TTGGGTGGAGTTTCGTAATTT‐3′). A scramble control siRNA (sequence: 5′‐ATTGAACAGAGGTTGTATATG‐3′) was used for the control group. Transfection of 50 nM siRNA into cells was performed using Lipofectamine RNAiMAX (Invitrogen) according to the manufacturer's instructions.

### 2.6 CCK‐8 Cell Viability Assay

HP75 cells were seeded in 96‐well plates (Corning Costar, 3599) at a density of 1 × 10^5^ cells/well (100 μL/well). Upon reaching approximately 80% confluence, 10 μL of CCK‐8 working solution (Dojindo Laboratories, CK04‐500) was added to each well, followed by incubation in the dark for 2 h. Absorbance was measured at a wavelength of 450 nm using a microplate reader. Cell viability was expressed as a percentage relative to the control group.

### 2.7 EdU Incorporation Assay

Cells were seeded in 96‐well plates (Corning Costar, 3599) at 2 × 10^5^ cells/well and incubated with 50 μM EdU (RiboBio, Cell‐Light EdU Apollo567 In Vitro Imaging Kit, C10310‐1) for 2 h. Subsequent steps were performed according to the kit protocol: fixation with 4% paraformaldehyde (Beyotime, P0099, 30 min at room temperature), permeabilisation with 0.5% Triton X‐100 (Sigma‐Aldrich, T8787, 30 min at room temperature), Apollo staining and Hoechst counterstaining (each for 30 min, protected from light). Images were captured using an inverted fluorescence microscope, and the EdU‐positive rate was calculated using ImageJ.

### 2.8 Annexin‐V/PI Apoptosis Detection

Cells were seeded in 6‐well plates (Corning, 3516) at 2 × 10^5^ cells/well and harvested during the logarithmic growth phase. Cells were resuspended in 1× Binding Buffer, incubated with Annexin V‐FITC for 15 min in the dark, followed by incubation with PI for 5 min; all reagents were from the Annexin V‐FITC/PI Apoptosis Detection Kit (BD Biosciences, FITC Annexin V Apoptosis Detection Kit I, 556547, which includes 1× Binding Buffer). A minimum of 10,000 events were acquired using a flow cytometer, and the proportion of early and late apoptotic cells was analysed using FlowJo.

### 2.9 Cell Invasion and Migration Assay

Transwell chambers (8 μm pore size, for 24‐well plates, Corning, 3422) were used. For the invasion assay, the upper chamber was coated with Matrigel Basement Membrane Matrix (Corning, Growth Factor Reduced, LDEV‐free, 354234) diluted 1:20 in serum‐free DMEM (Gibco, 11965‐092) and incubated at 37°C for 4 h to allow gel formation; for the migration assay, uncoated upper chambers were used. Cells were resuspended in serum‐free DMEM and seeded into the upper chamber at 1 × 10^5^ cells/well; the lower chamber was filled with 800 μL of DMEM containing 10% fetal bovine serum (FBS, Gibco, 10099‐141). After incubation for 24 h at 37°C with 5% CO₂, non‐migrated/non‐invaded cells on the upper surface were gently removed using a cotton swab. Cells were fixed with 4% paraformaldehyde for 20 min (Beyotime, P0099) and stained with 0.1% crystal violet for 10 min (Beyotime, C0121), followed by washing with PBS (Beyotime, C0221A). Five random fields of view were selected and counted at 100× magnification under an inverted microscope, and the average count was calculated. Each experiment was independently repeated at least three times.

### 2.10 Quantitative Reverse Transcription Polymerase Chain Reaction (qRT‐PCR)

Total RNA was extracted from HP75 cells using the TRIzol method (Invitrogen, Thermo Fisher Scientific, 15596026). Concentration and purity were measured using a NanoDrop (Thermo Scientific NanoDrop 2000/2000c). One microgram of RNA was reverse transcribed into cDNA using a reverse transcription kit (PrimeScript RT reagent Kit with gDNA Eraser, Takara, RR047A) according to the manufacturer's instructions. qRT‐PCR was performed on the StepOnePlus platform using a SYBR Green system (TB Green Premix Ex Taq II (Tli RNaseH Plus), Takara, RR820A, 20 μL reaction volume) and analysed using a real‐time quantitative PCR instrument (StepOnePlus Real‐Time PCR System, Applied Biosystems) with the following programme: 95°C for 2 min; 40 cycles of 95°C for 15 s, 60°C for 30 s, 72°C for 30 s. Primers covering human/mouse NR3C1, AXIN2, c‐Myc and GAPDH were synthesised by Sangon Biotech. Each sample was run in triplicate, and independent experiments were performed at least three times. Relative expression levels were calculated using the 2^−ΔΔCt^ method with GAPDH as the internal reference control, and results are presented as mean ± SEM.

The primer sequences used for PCR were as follows:

Human primers: NR3C1 forward 5′‐ACAGCATCCCTTTCTCAACAG‐3′; NR3C1 reverse 5′‐AGATCCTTGGCACCTATTCCAAT‐3′; AXIN2 forward 5′‐CAACACCAGGCGGAACGAA‐3′; AXIN2 reverse 5′‐GCCCAATAAGGAGTGTAAGGACT‐3′; c‐Myc forward 5′‐GGCTCCTGGCAAAAGGTCA‐3′; c‐Myc reverse 5′‐CTGCGTAGTTGTGCTGATGT‐3′; GAPDH forward 5′‐GGAGCGAGATCCCTCCAAAAT‐3′; GAPDH reverse 5′‐GGCTGTTGTCATACTTCTCATGG‐3′.

Mouse primers: c‐Myc forward 5′‐ATGCCCCTCAACGTGAACTTC‐3′; c‐Myc reverse 5′‐GTCGCAGATGAAATAGGGCTG‐3′; AXIN2 forward 5′‐ATGAGTAGCGCCGTGTTAGTG‐3′; AXIN2 reverse 5′‐GGGCATAGGTTTGGTGGACT‐3′; GAPDH forward 5′‐AGGTCGGTGTGAACGGATTTG‐3′; GAPDH reverse 5′‐GGGGTCGTTGATGGCAACA‐3′.

Relative mRNA expression was evaluated using the 2^−ΔΔCt^ method, with GAPDH serving as the internal reference control for gene expression levels.

### 2.11 TOP/FOP Dual‐Luciferase Assay

HP75 cells were seeded in 24‐well plates (1 × 10^5^/well) and allowed to adhere overnight. Cells were co‐transfected using Lipofectamine 3000 (Thermo Fisher, L3000015) with either TOPflash (Addgene, #12456) or FOPflash (Addgene, #12457) (500 ng each) and the Renilla internal control vector (Promega, E2241, pRL‐TK, 50 ng), along with sh‐NC or sh‐NR3C1 as required. Cells were lysed 48 h post‐transfection, and luciferase activity was measured using the Promega Dual‐Luciferase Reporter Assay System (Promega, E1910). Firefly luciferase activity was normalised to Renilla luciferase activity (Firefly/Renilla), and the TOP/FOP ratio was reported. Experiments were independently repeated at least three times.

### 2.12 Immunohistochemistry (IHC)

Formalin‐fixed paraffin‐embedded (FFPE) tissue sections (4 μm) were deparaffinised in xylene and rehydrated through a graded ethanol series. Antigen retrieval was performed by microwave heating in citrate antigen retrieval buffer (pH 6.0, Beyotime, P0081). After natural cooling to room temperature and rinsing with distilled water, endogenous peroxidase activity was blocked with 3% H₂O₂ (Beyotime, ST037, 10 min at room temperature), followed by blocking with 5%–10% goat serum (Beyotime, C0265, 30 min at room temperature). Sections were incubated with primary antibody against NR3C1/GR (Cell Signalling Technology, CST, #3660, rabbit monoclonal, IHC 1:200) overnight at 4°C in a humidified chamber. The next day, sections were warmed to room temperature and incubated with an HRP‐polymer secondary antibody (ZSGB‐Bio, Zhongshan Golden Bridge, PV‐9001, anti‐rabbit) according to the manufacturer's instructions, followed by colour development with DAB (ZSGB‐Bio, ZLI‐9018) until the desired intensity was achieved; the reaction was stopped with distilled water. Sections were counterstained with haematoxylin (Beyotime, C0107), dehydrated through graded ethanol, cleared in xylene and mounted with neutral balsam mounting medium (Solarbio, G8590). Images were acquired using an optical microscope. For quantification, the H‐score or positive area percentage method was employed, and analysis was performed using ImageJ; results are expressed as mean ± SEM.

### 2.13 Western Blot

Cells were lysed using RIPA lysis buffer (Beyotime, P0013B, supplemented with protease inhibitor cocktail P1005 and phosphatase inhibitor cocktail P1081). Protein concentration was quantified using the BCA method (Thermo Scientific, 23225), and equal amounts of protein (20–30 μg) were loaded, denatured at 95°C for 5 min, separated by 10% SDS‐PAGE, and transferred onto PVDF membranes (0.45 μm, Millipore, Immobilon‐P, IPVH00010). Membranes were blocked with 5% non‐fat milk in TBST (Beyotime, P0231) for 1 h at room temperature, followed by incubation with primary antibodies overnight at 4°C: GR/NR3C1 (Cell Signalling Technology, CST, #3660, rabbit monoclonal, 1:1000), phospho‐GR‐Ser211 (CST, #4161, rabbit monoclonal, 1:1000), β‐catenin (CST, #8480, rabbit monoclonal, 1:1000), GSK3β (CST, #9315, rabbit monoclonal, 1:1000), MMP‐9 (CST, #13667, rabbit monoclonal, 1:1000), E‐cadherin (CST, #3195, rabbit monoclonal, 1:1000), N‐cadherin (CST, #13116, rabbit monoclonal, 1:1000), β‐actin (Abcam, ab8226, mouse monoclonal, 1:5000) and Lamin B1 (Abcam, ab133741, rabbit monoclonal, 1:5000). The next day, membranes were washed three times with TBST for 10 min each and incubated with HRP‐conjugated secondary antibodies for 1 h at room temperature (goat anti‐rabbit IgG, CST, #7074, 1:5000; goat anti‐mouse IgG, CST, #7076, 1:5000). After another three washes with TBST for 10 min each, blots were developed using ECL chemiluminescent substrate and imaged (Thermo Scientific, SuperSignal West Pico PLUS, 34580; using a system such as the Bio‐Rad ChemiDoc). Band intensity was quantified using ImageJ/Fiji or Bio‐Rad Image Lab software. Bands were first normalised to internal controls (β‐actin for total protein/cytoplasmic fractions, Lamin B1 for nuclear extracts; calculated as target protein intensity/internal control intensity), and then experimental groups were normalised relative to the control group set as 1 to obtain relative expression levels. Statistics and graphing were performed using GraphPad Prism 9, and results are presented as mean ± SEM. All experiments were independently repeated at least three times.

### 2.14 Nude Mouse Xenograft Tumour Model

A nude mouse xenograft tumour model was established to study the in vivo tumorigenic ability of TtT/GF cells. Eighteen nude mice were randomly divided into three experimental groups, with six mice per group (*n* = 6). The groups included: sh‐NC + DMSO, sh‐NR3C1 + DMSO and sh‐NR3C1 + CHIR99021. CHIR99021 is a highly specific GSK3β inhibitor that activates canonical Wnt signalling by stabilising β‐catenin, a key mediator of the pathway. TtT/GF cells were cultured under standard conditions and harvested during the logarithmic growth phase. Cells were then suspended in sterile phosphate‐buffered saline (PBS) at a concentration of 1 × 10^6^ cells per 100 μL. Cell suspensions were injected subcutaneously into the right flank of each mouse. For the CHIR99021 treatment group, the Wnt pathway agonist CHIR99021 was administered via intraperitoneal injection at a dose of 10 mg/kg every 2 days for 2 weeks or until the experimental endpoint. The solvent for CHIR99021 consisted of a mixture of DMSO, PEG400 and saline in a 1:3:6 ratio. Tumour growth was monitored every 2 weeks by measuring tumour length (*L*) and width (*W*) with callipers. Tumour volume (*V*) was calculated using the formula *V* = (*L* × *W* ^2^)/2. Tumour growth curves were plotted to compare tumour progression among different groups. At the end of the treatment period, mice were euthanised, tumours were excised, weighed and photographed to document their size and morphology. For histological analysis, tumour tissues were fixed in 10% formalin, paraffin‐embedded, sectioned and stained with haematoxylin and eosin (H&E). Stained sections were examined under a microscope for histopathological features and morphological changes induced by the treatments.

### 2.15 Statistical Analysis

Statistical analysis of all data was performed using R software (version 4.0.3) and SPSS software (version 26.0). Differential expression analysis of RNA‐seq data and proteomics data was conducted using the DESeq2 and limma packages, respectively. For RNA‐seq data, criteria of |log2 fold change| > 1 and adjusted *p*‐value < 0.05 were used to screen for DEGs. Analysis of mass spectrometry proteomics data was performed using MaxQuant software, with screening criteria set at |log2 fold change| > 1 and *p*‐value < 0.05. Gene Set Enrichment Analysis (GSEA) was performed using GSEA software to evaluate the activation status of pathways associated with high expression of target genes. For GSEA analysis, a Nominal *p*‐value < 0.05 and FDR < 0.25 were set as significance criteria. Comparisons between groups were performed using independent sample *t*‐tests or Mann–Whitney U tests, with a *p*‐value < 0.05 considered statistically significant. Receiver operating characteristic (ROC) curve analysis was used to evaluate the diagnostic efficacy of NR3C1, calculating the area under the curve (AUC) and performing DeLong's test. All experimental data are presented as mean ± standard deviation (Mean ± SD), and all statistical tests were two‐sided.

## 3 Results

### 3.1 NR3C1 Is a Potential Key Gene in Non‐Functional Invasive PAs

**FIGURE 1:**
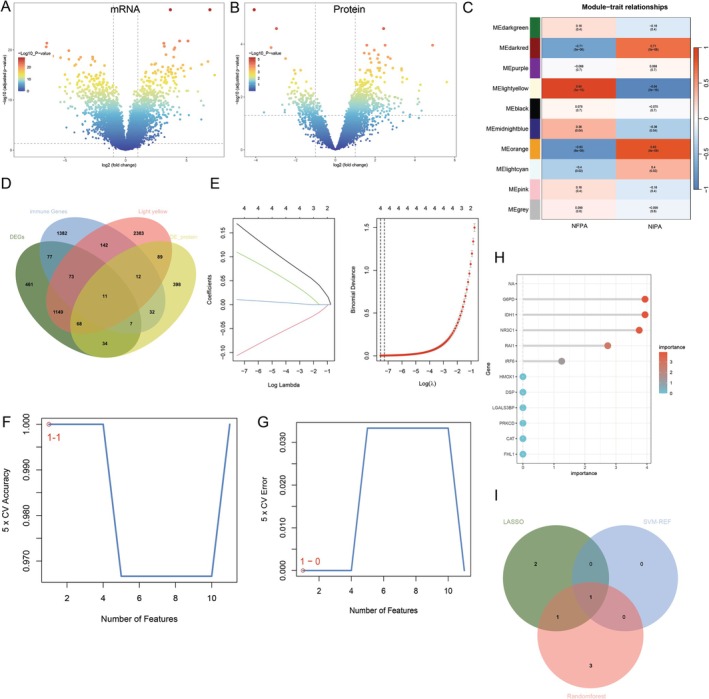
Multi‐omics analysis and experimental validation of NR3C1 in NIPAs. (A) Volcano plot of differentially expressed genes (DEGs) from mRNA sequencing. DEGs were screened with thresholds |log_2_(fold change)| > 1 and false discovery rate (FDR) < 0.05. (B) Volcano plot of differentially expressed proteins from mass spectrometry. Differential proteins were filtered with |log_2_(fold change)| > 1 and *p* < 0.05. (C) Heatmap depicting the correlation between Weighted Gene Co‐expression Network Analysis (WGCNA) modules and the traits non‐functioning invasive pituitary adenomas (NIPAs) and non‐functioning non‐invasive pituitary adenomas (NFPAs). The ME light yellow module showed the strongest positive correlation with NIPAs (*r* = 0.94, *p* < 0.01). (D) Venn diagram showing the intersection of four datasets: Transcriptomic DEGs, proteomic differential proteins, genes from WGCNA MElightyellow module, and 1736 immune‐related genes retrieved from GeneCards. This intersection yielded 11 candidate genes closely linked to immunity and invasiveness. (E) LASSO regression analysis for feature selection: Left panel shows the regularisation path of gene coefficients (each line represents one gene), right panel shows the cross‐validation curve. The optimal *λ* value (*λ* = 0.05) was determined by minimising cross‐validation error, retaining four feature genes including NR3C1. (F) Plot of classification accuracy versus the number of features under fivefold cross‐validation (5‐CV). The *x*‐axis shows the number of features and the *y*‐axis shows the 5‐CV classification accuracy. The ‘1–1’ label marks the peak accuracy at the optimal feature count. (G) Plot of classification error versus the number of features under fivefold cross‐validation. The *x*‐axis shows the number of features and the *y*‐axis shows the 5‐CV classification error. The ‘1–1’ label marks the minimum error at the optimal feature count. (H) Random Forest (RF) feature importance ranking (mean decrease Gini coefficient). A threshold of importance score > 1 was set for strict feature screening, resulting in five candidate genes. NR3C1 was identified as one of these five high‐importance genes. (I) Venn diagram of the intersection of three machine learning results (LASSO regression, SVM‐RFE, RF). NR3C1 was the only gene consistently identified by all three algorithms.

To investigate the key genes and underlying mechanisms in NIPAs, we conducted a stepwise multi‐omics analysis to identify genes playing significant roles in NIPAs. Firstly, we performed RNA‐seq analysis on 32 pituitary adenoma samples to establish mRNA expression profiles. Figure 1A shows the volcano plot of significantly DEGs. We also established a proteomic database from 19 pituitary adenoma samples via mass spectrometry, with Figure 1B displaying the volcano plot of significantly differentially expressed proteins. Subsequently, we employed WGCNA to identify gene modules associated with pituitary adenoma invasion. Figure 1C presents a heatmap showing the correlation of different modules with NIPAs and NFPAs; the MElightyellow module showed the strongest positive correlation with NIPAs (*r* = 0.94, *p* < 0.01), defining it as the key module for further analysis. To further screen for DEGs related to immunity, we obtained 1736 immune‐related DEGs from the GeneCards database, and a Venn diagram (Figure 1D) illustrates the intersection of these immune‐related genes, RNA‐seq DEGs and genes in the WGCNA MElightyellow module, yielding 11 candidate genes. We then applied multiple machine learning algorithms (including LASSO regression, SVM‐RFE and RF) to screen for target genes closely associated with both immunity and invasiveness. Figure 1E shows the LASSO regression path plot (left, gene coefficient shrinkage with increasing *λ*) and cross‐validation curve (right, identifying the optimal *λ* = 0.05 to minimise error). Figure 1F displays the fivefold cross‐validation (CV) accuracy curve (*y*‐axis = accuracy, *x*‐axis = number of retained features), while Figure 1G shows the fivefold CV error curve (*y*‐axis = error rate, *x*‐axis = number of retained features). Figure 1H shows the RF feature importance scores (mean decrease Gini coefficient), with a threshold of importance score > 1 set for screening, resulting in five high‐importance genes including NR3C1. Figure 1I shows the intersection of genes obtained from the three machine learning methods, confirming NR3C1 as the sole consensus candidate gene for subsequent functional validation.

**FIGURE 2:**
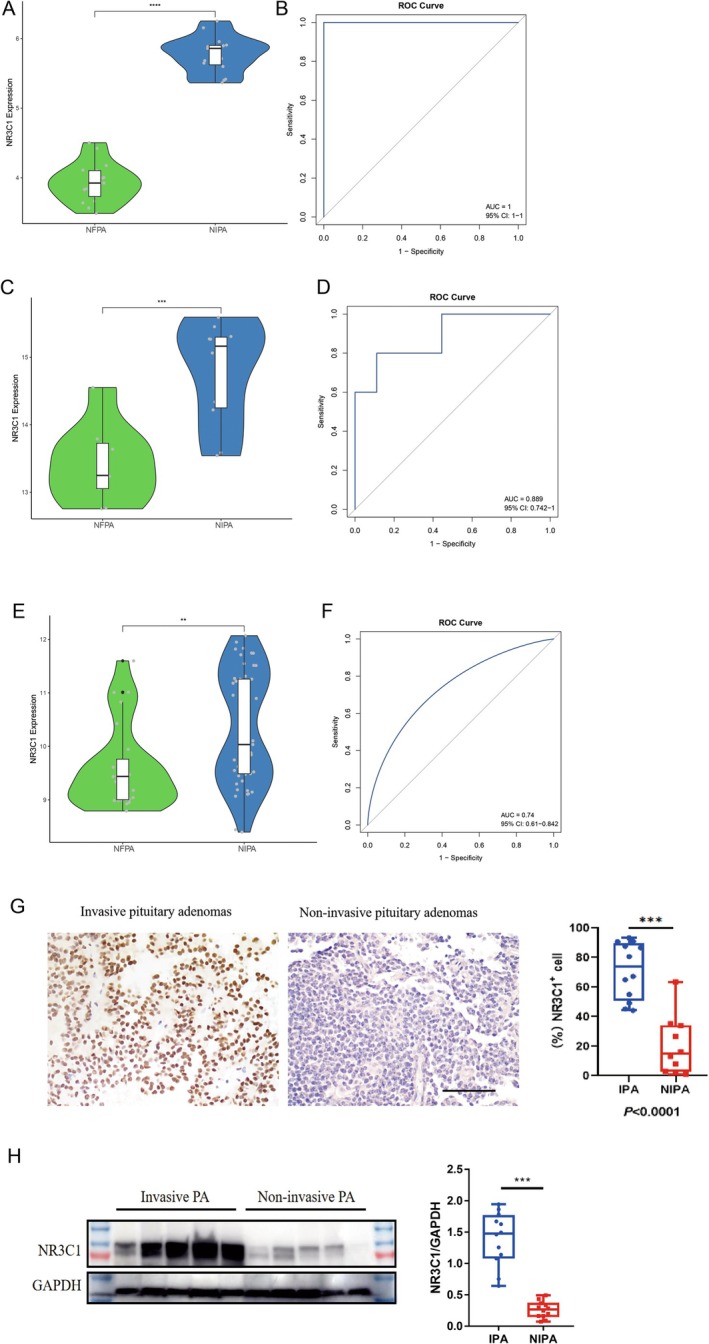
Elevated expression of NR3C1 in non‐functional invasive pituitary adenomas. (A) Expression levels of NR3C1 in NIPAs and NFPAs groups from RNA‐seq data. (B) Corresponding receiver operating characteristic (ROC) curve for NR3C1 expression from RNA‐seq data. (C) Expression levels of NR3C1 in NIPAs and NFPAs groups from the proteomics dataset. (D) Corresponding ROC curve for NR3C1 expression from the proteomics dataset. (E) Expression levels of NR3C1 in the external validation set (GSE169498) comprising NIPAs and NFPAs samples. (F) Corresponding ROC curve for NR3C1 expression in the external validation set comprising NIPAs and NFPAs samples. (G) Immunohistochemical (IHC) analysis of NR3C1 in clinical samples, showing the percentage of NR3C1‐positive cells in the NIPAs and NFPAs groups. (H) Western blot analysis of NR3C1 protein expression levels in clinical samples (*p* < 0.0001, ** *p* < 0.01, *** *p* < 0.001, **** *p* < 0.0001).

To further validate the expression and role of NR3C1 in NIPAs, we performed validation through multi‐omics data. Firstly, RNA‐seq analysis revealed that NR3C1 expression was significantly higher in NIPAs compared to NFPAs. Figure 2A shows the expression levels of NR3C1 in the NIPAs and NFPAs groups, and Figure 2B displays the corresponding receiver operating characteristic (ROC) curve, showing an AUC value of 1.0, indicating NR3C1's high ability to distinguish between NIPAs and NFPAs. In the proteomic dataset, we similarly found high expression of NR3C1 in NIPAs. Figure 2C,D show the protein‐level expression of NR3C1 and its corresponding ROC curve (AUC = 0.889, 95% CI: 0.742–1.0), respectively. In an external validation set (GSE169498), NR3C1 expression was also significantly higher in NIPAs compared to NFPAs. Figure 2E shows NR3C1 expression in this dataset, and Figure 2F shows the ROC curve for this dataset (AUC = 0.74, 95% CI: 0.661–0.842), further supporting our findings. To further validate NR3C1 expression in clinical samples, we performed immunohistochemical analysis (Figure 2G) and western blot analysis (Figure 2H) comparing NR3C1 expression between invasive and non‐invasive PAs. The results showed that NR3C1 protein expression levels were significantly higher in the NIPAs group compared to the NFPAs group. These results fully demonstrate the high expression of NR3C1 in NIPAs, supporting its potential as a diagnostic biomarker and therapeutic target.

### 3.2 Association of NR3C1 With Classical Immune Checkpoint Genes in the Transcriptome

**FIGURE 3:**
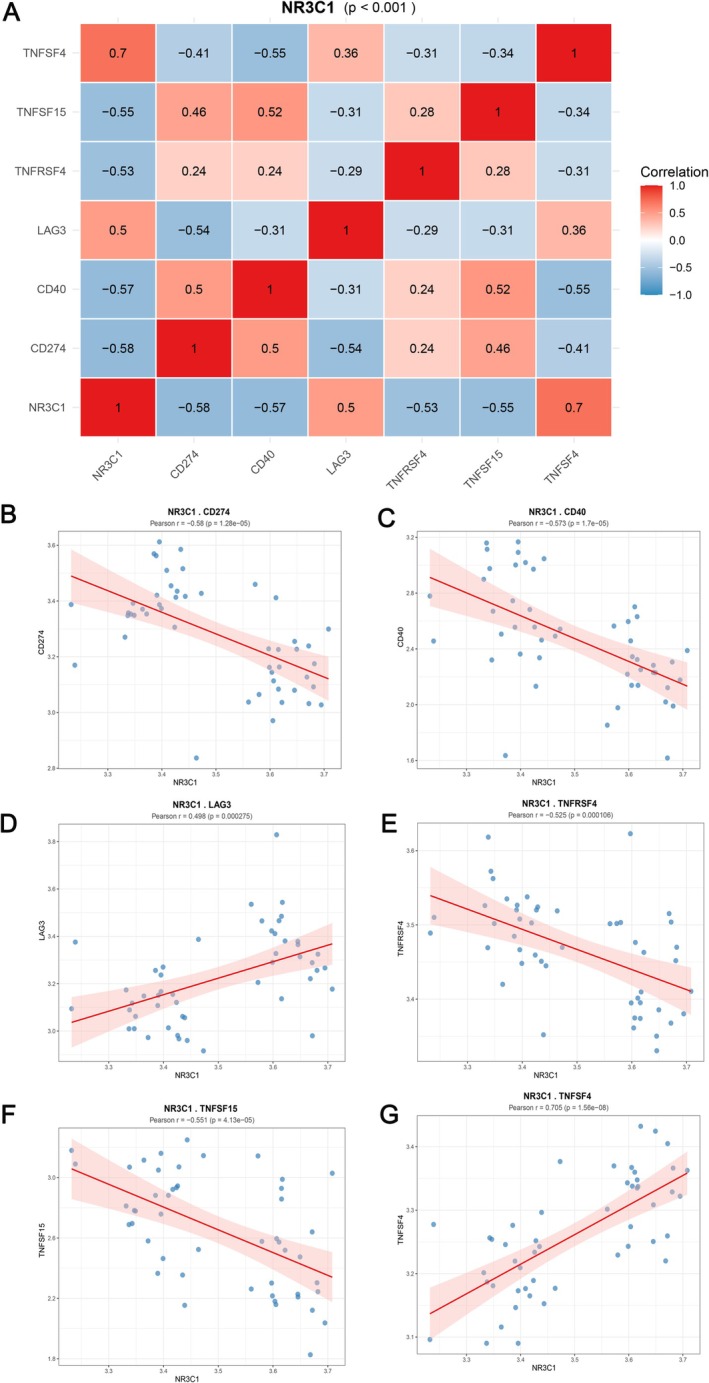
Correlation analysis between NR3C1 and classical immune checkpoint genes. (A) Heatmap of correlations between NR3C1 and multiple immune checkpoint genes; red indicates positive correlation, blue indicates negative correlation. (B) Negative correlation between NR3C1 and CD274 expression (Pearson *r* = −0.58, *p*‐value = 3.16e‐05). (C) Negative correlation between NR3C1 and CD40 expression (Pearson *r* = −0.57, *p*‐value = 1.17e‐05). (D) Negative correlation between NR3C1 and LAG3 expression (Pearson *r* = −0.49, *p*‐value = 0.0002275). (E) Negative correlation between NR3C1 and TNFRSF4 expression (Pearson *r* = −0.52, *p*‐value = 0.0001676). (F) Negative correlation between NR3C1 and TNFSF15 expression (Pearson *r* = −0.51, *p*‐value = 1.47e‐05). (G) Positive correlation between NR3C1 and TNFSF4 expression (Pearson *r* = 0.70, *p*‐value = 1.56e‐06).

To explore the association between NR3C1 and classical immune checkpoint genes, we performed correlation analysis using transcriptomic data. First, the expression correlation between NR3C1 and 47 classical immune checkpoint genes was calculated to reveal its potential role in NIPAs. Correlation analysis revealed that NR3C1 had significant correlations with multiple immune checkpoint genes. Figure 3A shows a heatmap of the correlations between NR3C1 and the immune checkpoint genes, where red indicates positive correlation and blue indicates negative correlation. Further analysis showed that NR3C1 had significant negative or positive correlations with the expression levels of genes such as CD274, CD40, LAG3, TNFRSF4, TNFSF15 and TNFSF4. To further confirm these correlations, scatter plots of NR3C1 against selected immune checkpoint genes were generated. The results showed that NR3C1 was significantly negatively correlated with CD274 (Pearson *r* = −0.58, *p*‐value = 3.16e‐05), significantly negatively correlated with CD40 (Pearson *r* = −0.57, *p*‐value = 1.17e‐05), significantly negatively correlated with LAG3 (Pearson *r* = −0.49, *p*‐value = 0.0002275), significantly negatively correlated with TNFRSF4 (Pearson *r* = −0.52, *p*‐value = 0.0001676), significantly negatively correlated with TNFSF15 (Pearson *r* = −0.51, *p*‐value = 1.47e‐05), and significantly positively correlated with TNFSF4 (Pearson *r* = 0.70, *p*‐value = 1.56e‐06) (Figure 3B–G). The expression of NR3C1 showed significant correlations with the expression levels of various classical immune checkpoint genes. These findings suggest that NR3C1 expression is correlated with the expression of classical immune checkpoint genes in NIPAs, indicating a potential indirect association with the tumour immune microenvironment.

### 3.3 Association of NR3C1 With Immune Cell Infiltration in Non‐Functional Invasive PAs

**FIGURE 4:**
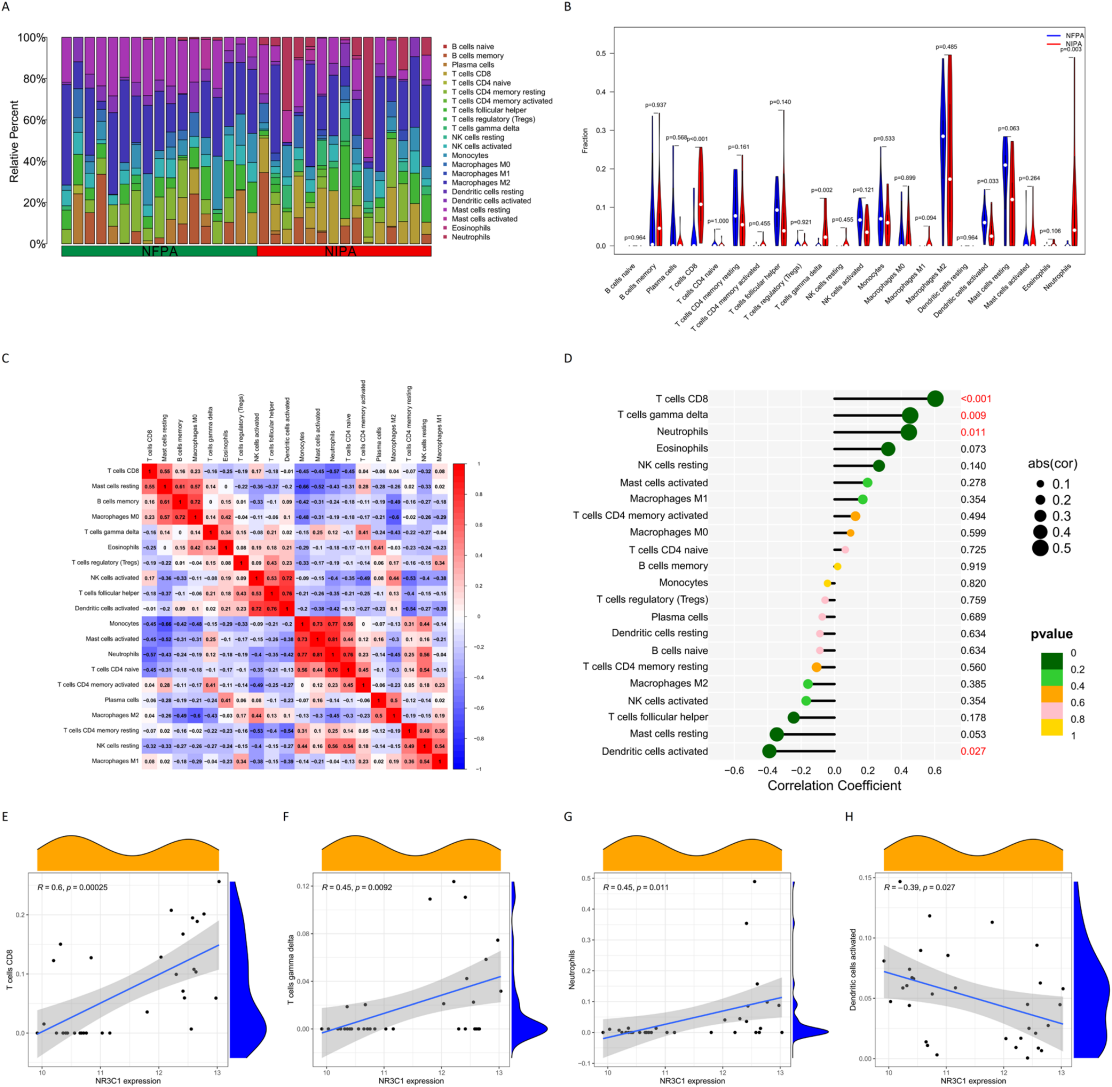
Correlation analysis between NR3C1 and immune cell infiltration. (A) Relative infiltration proportions of various immune cell types in NIPAs and NFPAs. (B) Differential infiltration levels of each immune cell type between NIPAs and NFPAs; significantly different cell types (*p* < 0.05) are marked. (C) Correlation heatmap among different immune cell types; red and blue indicate positive and negative correlations, respectively. (D) Correlation between NR3C1 expression and various immune cell types; significant relationships are marked in red (negative) and green (positive). (E) Positive correlation between NR3C1 expression and CD8^+^ T cell infiltration. (F) Positive correlation between NR3C1 expression and gamma delta T cell infiltration. (G) Positive correlation between NR3C1 expression and M0 macrophage infiltration. (H) Positive correlation between NR3C1 expression and activated dendritic cell infiltration.

To investigate the role of NR3C1 in NIPAs, we conducted a comprehensive analysis of immune cell infiltration. Figure 4A shows the relative infiltration proportions of various immune cell types in NIPAs and NFPAs. Compared to NFPAs, NIPAs exhibited distinctly different patterns of immune cell infiltration, with specific immune cell types such as T cells, macrophages and natural killer (NK) cells showing significant differences between the two types of pituitary tumours. In further analysis of the differences in infiltration of these immune cells, Figure 4B displays the differential expression (infiltration levels) of each immune cell type in NFPAs versus NIPAs. Significantly affected immune cells included CD8^+^ T cells, gamma delta T cells and activated NK cells, all of which were highly expressed (infiltrated) in NIPAs and relatively low in NFPAs. Statistical analysis indicated that these differences in cell infiltration were significant (*p* < 0.05). To gain insight into the correlations between these immune cell types, we generated a correlation heatmap (Figure 4C), showing the interrelationships among different immune cell categories. The red and blue squares in the heatmap represent positive and negative correlations, respectively, further revealing the complex relationships between cells. Furthermore, we validated the relationship between NR3C1 expression and immune cell infiltration. Figure 4D shows the correlation between NR3C1 and various immune cell types, where negative and positive correlations are marked in red and green, respectively. Significantly correlated immune cells included CD8^+^ T cells (*p* < 0.001), gamma delta T cells (*p* < 0.01), M0 macrophages (*p* < 0.05) and activated dendritic cells (*p* < 0.05). Further scatter plot analysis (Figure 4E–H) shows the correlation between NR3C1 expression and the infiltration levels of several significant immune cell types (CD8^+^ T cells, gamma delta T cells, M0 macrophages, activated dendritic cells). The results indicate a significant positive correlation between NR3C1 expression levels and the extent of infiltration of these immune cells. These findings suggest that NR3C1 expression shows a significant positive correlation with the infiltration of specific immune cell types, suggesting a potential link between NR3C1‐driven tumour invasiveness and immune cell recruitment.

### 3.4 The Impact of NR3C1 on the Biological Behaviour of Pituitary Adenoma Cells

**FIGURE 5:**
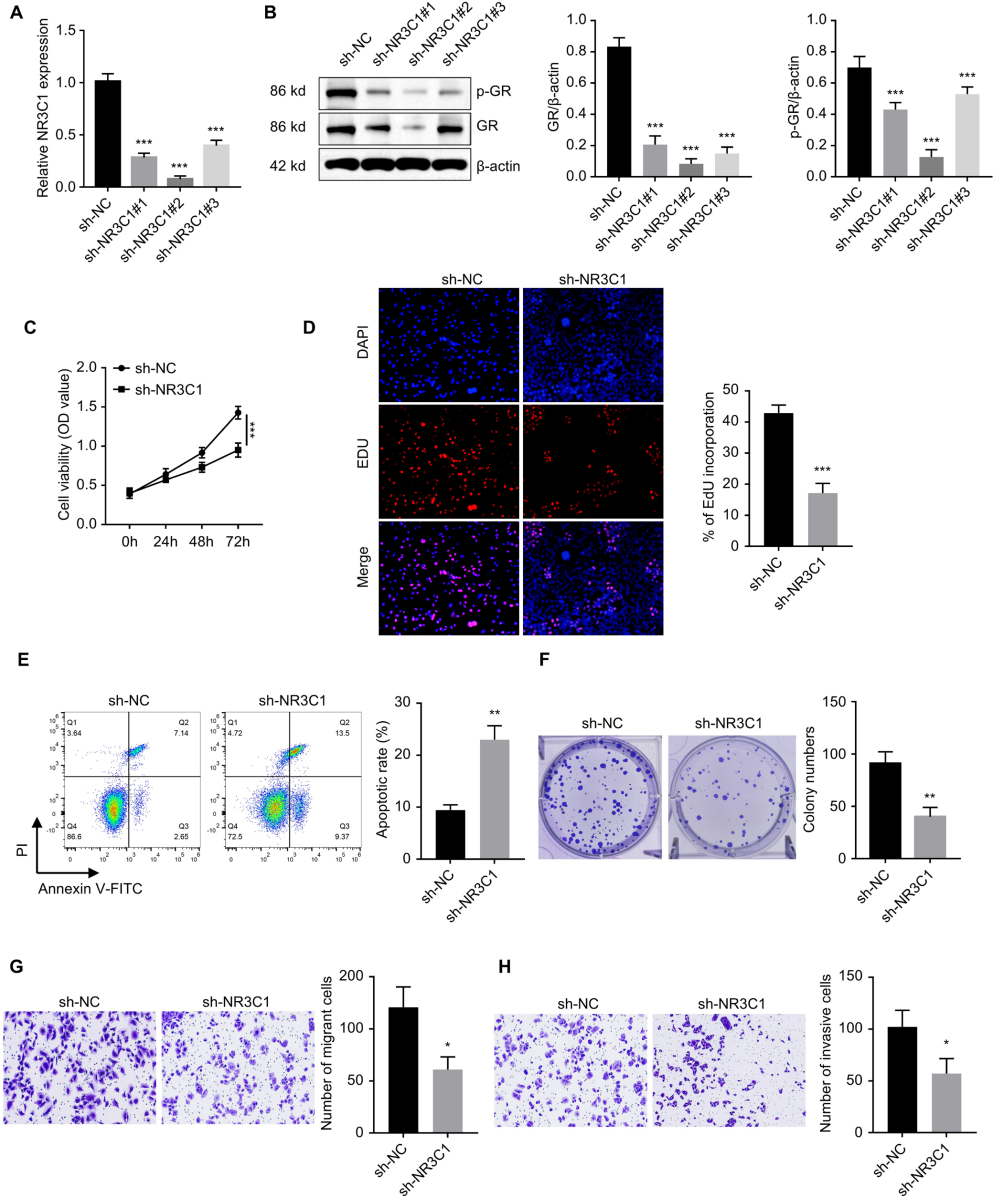
NR3C1 knockdown inhibits proliferation, clonogenicity, migration and invasion, and promotes apoptosis in pituitary adenoma cells. (A) NR3C1 gene knockdown efficiency mediated by RNA interference; three specific shRNAs significantly reduced NR3C1 mRNA expression. (B) Western blot analysis showing significantly reduced NR3C1 protein expression and phosphorylation levels. (C) CCK‐8 assay showing NR3C1 knockdown significantly inhibited cell proliferation capacity. (D) EdU incorporation assay showing NR3C1 knockdown significantly reduces the cell proliferation rate. (E) Annexin V‐PI double staining assay showing NR3C1 knockdown significantly increases the cell apoptosis rate. (F) Colony formation assay showing NR3C1 knockdown significantly inhibited cell clonogenic ability. (G) Transwell migration assay showing NR3C1 knockdown significantly inhibits cell migration ability. (H) Transwell invasion assay showing NR3C1 knockdown significantly inhibits cell invasion ability. **p < 0.01, ***p < 0.001.

To validate the effect of NR3C1 downregulation on the biological behaviour of pituitary adenoma cells, we conducted a series of functional experiments using the human pituitary adenoma cell line HP75. First, NR3C1 expression was downregulated using RNA interference technology. Figure 5A shows the knockdown efficiency of NR3C1 mediated by shRNA in HP75 cells; three specific shRNAs targeting NR3C1 significantly reduced NR3C1 mRNA levels (*p* < 0.001). Furthermore, Western blot analysis (Figure 5B) demonstrated that NR3C1 knockdown led to a significant decrease in its protein expression and a marked reduction in its phosphorylation level (*p* < 0.001). Next, we assessed cell viability. The CCK‐8 assay results showed that NR3C1 knockdown significantly inhibited HP75 cell proliferation (Figure 5C, *p* < 0.001). Similarly, EdU assay results (Figure 5D) also indicated a significant decrease in cell viability following NR3C1 knockdown (*p* < 0.01). Annexin V‐PI double staining revealed that NR3C1 knockdown significantly increased the apoptosis rate (Figure 5E, *p* < 0.001). Figure 5F shows that NR3C1 knockdown significantly inhibited the clonogenic ability of pituitary adenoma cells (*p* < 0.01). Finally, Transwell migration and invasion assays demonstrated that NR3C1 knockdown significantly suppressed HP75 cell migration (Figure 5G, *p* < 0.01) and invasion (Figure 5H, *p* < 0.01) capabilities. These results further support the critical role of NR3C1 in pituitary adenoma progression.

**FIGURE 6:**
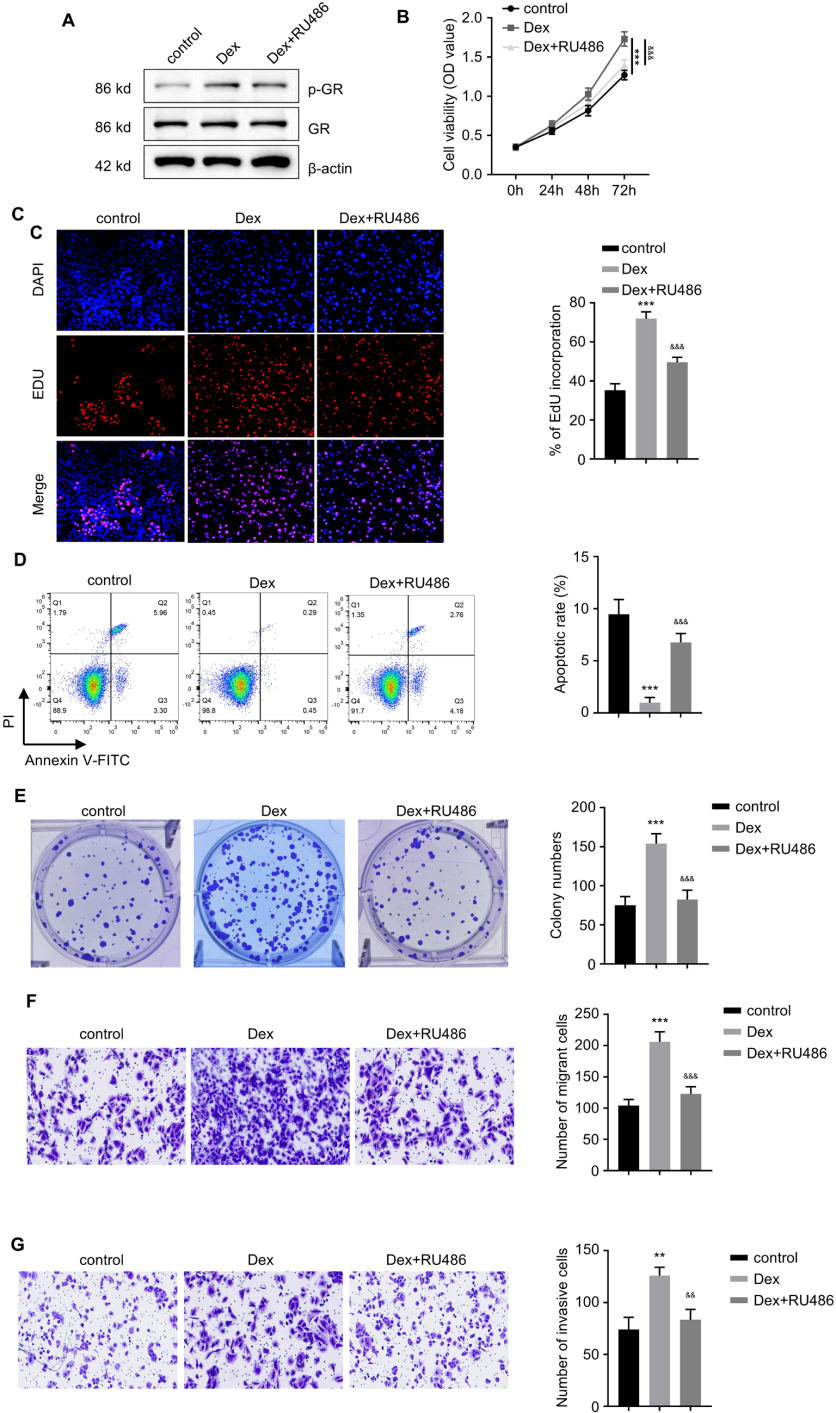
Dexamethasone (Dex) activates NR3C1 (GR) to promote proliferation, clonogenicity, migration and invasion and inhibit apoptosis in pituitary adenoma cells; mifepristone (RU486) antagonises NR3C1 and exerts opposite effects. (A) Western blot analysis showing the effects of Dex treatment and combined RU486 treatment on the expression of NR3C1 (GR) and its phosphorylated form (p‐GR). (B) CCK‐8 assay evaluating the effects of Dex and RU486 on cell proliferation capacity. (C) EdU incorporation assay showing Dex treatment significantly increased cell proliferation capacity, while RU486 inhibition significantly reduced this capacity. (D) Annexin V‐FITC‐based apoptosis detection showing Dex treatment inhibited apoptosis, while RU486 treatment significantly increased the apoptosis rate. (E) Colony formation assay evaluating the effects of Dex and RU486 on cell clonogenic ability. (F) Transwell migration assay showing Dex significantly promoted cell migration while RU486 treatment significantly inhibited this process. (G) Transwell invasion assay showing Dex significantly enhanced cell invasion ability, while RU486 treatment significantly inhibited cell invasion. **p < 0.01, ***p < 0.001, &&&, *p* < 0.001 compared with Dex group.

To verify the effects of dexamethasone (Dex) and the NR3C1 inhibitor RU486 on pituitary adenoma cells, we performed Western blot analysis using HP75 cells (Figure 6A). The results showed that Dex treatment significantly increased the expression of p‐GR (phosphorylated NR3C1), whereas RU486 treatment markedly reduced this expression level, indicating that NR3C1 activation was induced by Dex and inhibited by RU486.

All subsequent experiments in this subsection (CCK‐8, EdU, apoptosis, colony formation, Transwell assays) were explicitly linked to HP75 cells. In cell proliferation experiments, we assessed cell viability using the CCK‐8 kit (Figure 6B). The results indicated that Dex treatment significantly promoted cell proliferation, while combined treatment with RU486 significantly inhibited proliferation (*p* < 0.001). This finding was further validated by the EdU incorporation assay (Figure 6C), where NR3C1 activation promoted cell proliferation, and its inhibition significantly reduced the cell proliferation rate (*p* < 0.01). Apoptosis was detected via Annexin V‐FITC staining (Figure 6D). The experimental results showed that Dex treatment significantly decreased the cell apoptosis rate, whereas combined RU486 treatment significantly increased the apoptosis rate (*p* < 0.001), indicating that NR3C1 inhibition promotes apoptosis. The colony formation assay results (Figure 6E) demonstrated that Dex treatment significantly enhanced the clonogenic ability of pituitary adenoma cells, while the addition of RU486 significantly inhibited this ability (*p* < 0.001). This further supports the important role of NR3C1 in cell proliferation and survival. Finally, we assessed cell invasive capability using Transwell migration and invasion assays (Figure 6F,G). The results showed that Dex treatment significantly increased cell migration and invasion abilities, while combined RU486 treatment led to a significant decrease in these capabilities (*p* < 0.001). The results suggest that NR3C1 activation promotes these cancerous behaviours, while its inhibition significantly suppresses this process.

### 3.5 The Role of NR3C1 in Non‐Functional Invasive PAs via the Wnt Signalling Pathway

**FIGURE 7:**
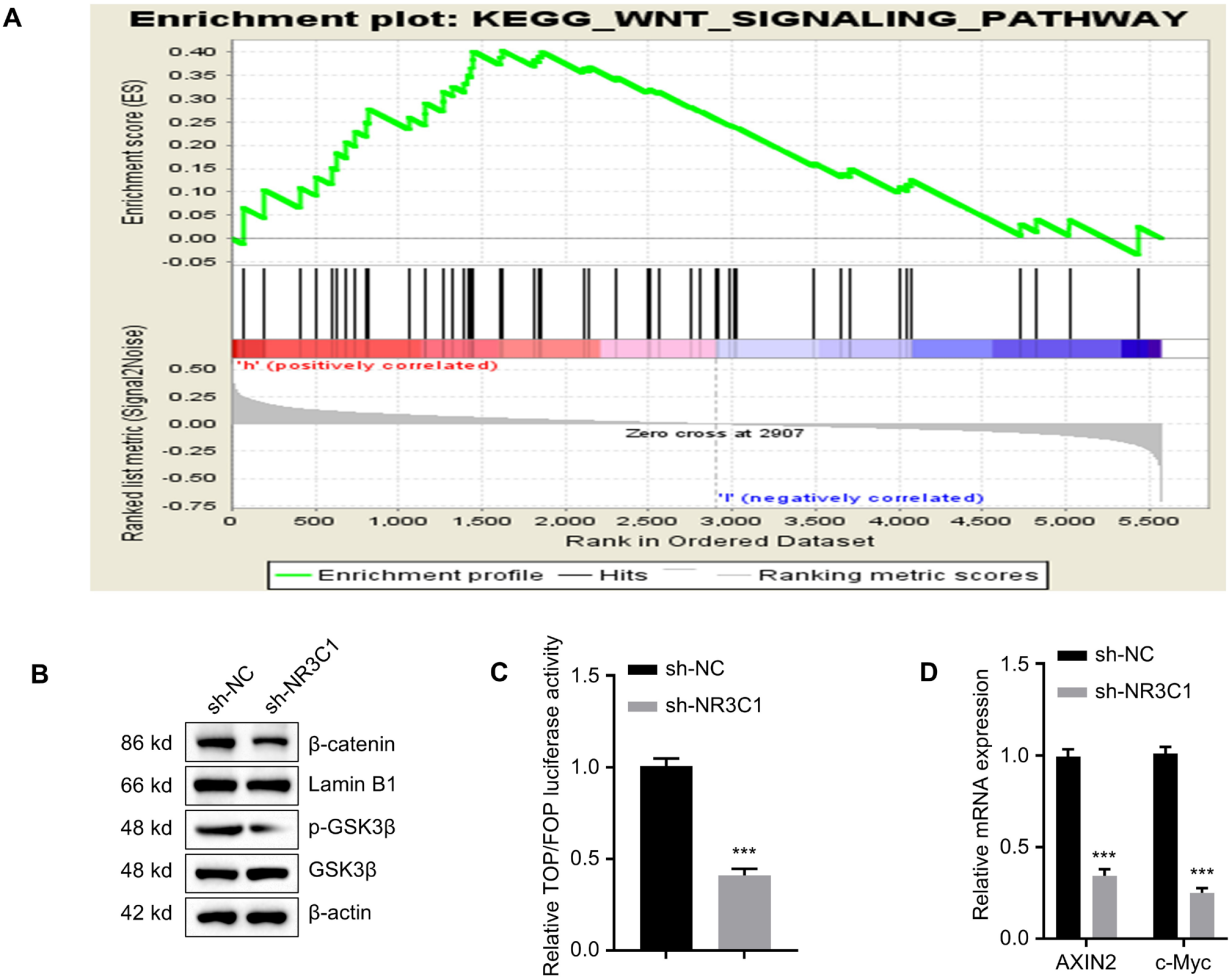
NR3C1 influences NIPAs by activating the Wnt signalling pathway. (A) Gene set enrichment analysis (GSEA) showing high NR3C1 expression is significantly associated with the Wnt signalling pathway. (B) Western blot analysis showing significantly reduced expression levels of key Wnt signalling pathway proteins after NR3C1 knockdown. (C) TOP/FOP luciferase reporter assay evaluating the effect of NR3C1 on Wnt signalling pathway activity; NR3C1 knockdown significantly reduced activity. (D) qPCR analysis showing NR3C1 knockdown significantly reduced the mRNA expression of Wnt pathway downstream target genes AXIN2 and c‐Myc. **p < 0.01, ***p < 0.001.

To investigate the mechanism of NR3C1 in NIPAs, we conducted GSEA and subsequent functional validation experiments in HP75 cells. GSEA of proteomics data revealed a significant positive correlation between NR3C1 and the Wnt signalling pathway (Figure 7A). This analysis suggested that high expression of NR3C1 might influence the invasiveness of PAs by activating the Wnt signalling pathway. Next, we validated the impact of NR3C1 on key proteins of the Wnt pathway via Western blot analysis (Figure 7B). The results showed that the expression levels of β‐catenin and p‐GSK3β were significantly reduced in the NR3C1 knockdown group (sh‐NR3C1), further supporting the GSEA results. TOP/FOP dual‐luciferase assay (Figure 7C) was performed in HP75 cells to evaluate Wnt pathway activity after NR3C1 knockdown. The results indicated that NR3C1 knockdown significantly reduced Wnt signalling pathway activity compared to the control group (sh‐NC) (*p* < 0.001). Using qPCR analysis, we detected the mRNA expression levels of the Wnt pathway downstream target genes AXIN2 and c‐Myc (Figure 7D). The results showed that NR3C1 knockdown significantly reduced the expression of AXIN2 and c‐Myc (*p* < 0.001), further validating that NR3C1 functions through the Wnt signalling pathway. These findings elucidate the mechanism of NR3C1 in NIPAs, providing new insights into its potential as a diagnostic biomarker and therapeutic target.

**FIGURE 8:**
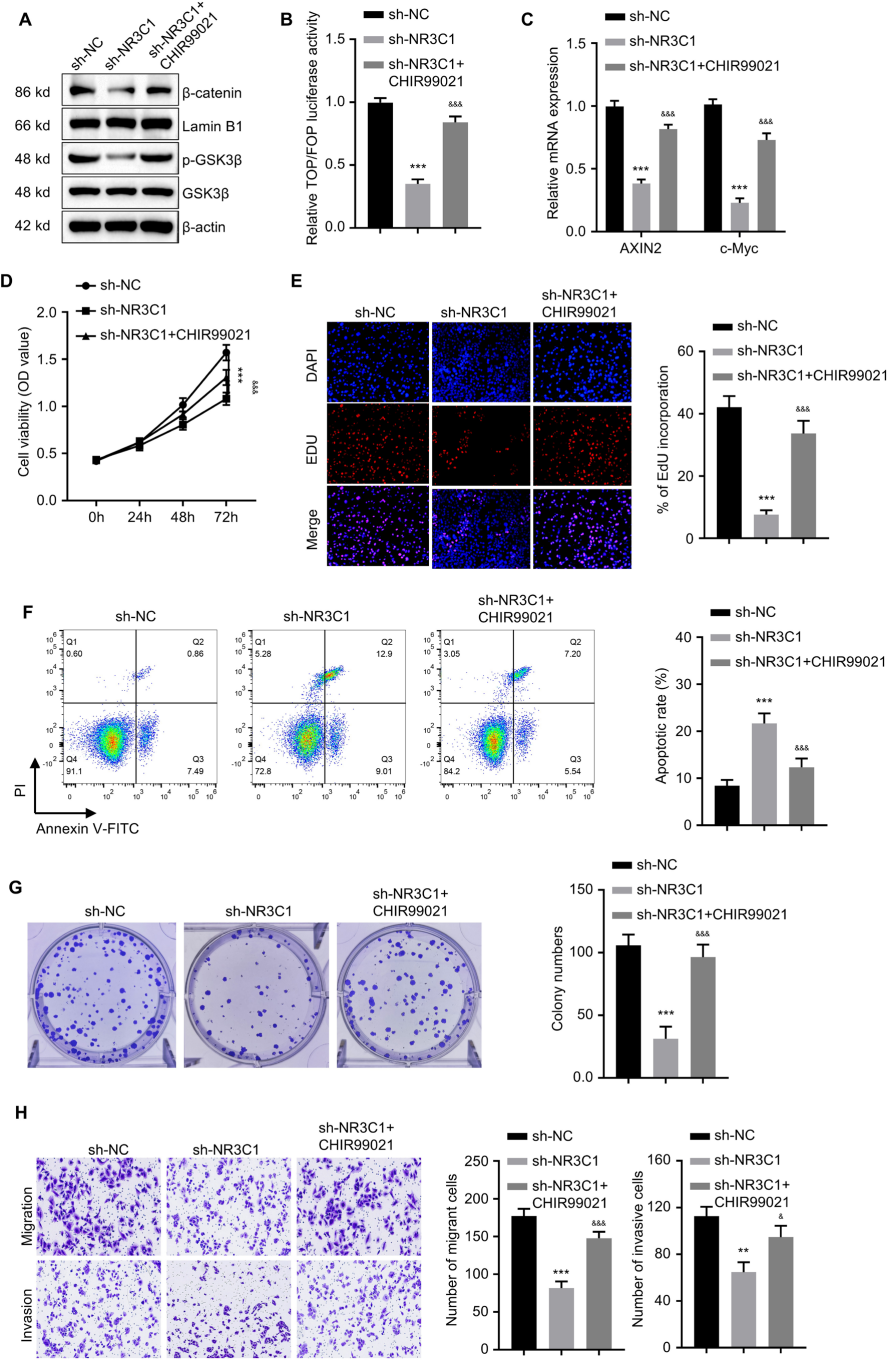
NR3C1 affects the biological behaviour of NIPA cells by activating the Wnt signalling pathway. (A) Western blot analysis showing the effect of NR3C1 knockdown on the expression of key proteins in the Wnt signalling pathway; addition of the GSK3β inhibitor CHIR99021 partially restored their expression. (B) TOP/FOP luciferase reporter assay showing NR3C1 knockdown significantly reduced Wnt pathway activity, and addition of CHIR99021 significantly rescued this effect (*p* < 0.001). (C) qPCR analysis showing NR3C1 knockdown significantly reduced mRNA expression of Wnt downstream target genes AXIN2 and c‐Myc, and addition of CHIR99021 partially restored this expression (*p* < 0.001). (D) CCK‐8 assay showing NR3C1 knockdown significantly inhibited cell proliferation capacity, which was partially restored after adding CHIR99021 (*p* < 0.001). (E) EdU incorporation assay showing NR3C1 knockdown significantly reduced the cell proliferation rate, and addition of CHIR99021 significantly restored proliferative capacity (*p* < 0.001). (F) Annexin V‐FITC‐based detection showing NR3C1 knockdown significantly increased the apoptosis rate, which was significantly reduced after adding CHIR99021 (*p* < 0.001). (G) Colony formation assay showing NR3C1 knockdown significantly inhibited cell clonogenic ability, which was significantly restored after adding CHIR99021 (*p* < 0.001). (H) Transwell migration and invasion assays showing NR3C1 knockdown significantly inhibited cell migration and invasion abilities, and addition of CHIR99021 significantly increased these capabilities (*p* < 0.001). **p < 0.01, ***p < 0.001, &&&, *p* < 0.001 compared with sh‐NR3C1.

GSK3β is a negative regulator of the canonical Wnt signalling pathway; under basal conditions, it promotes the degradation of β‐catenin. The selective GSK3β inhibitor CHIR99021 stabilises β‐catenin, thereby activating Wnt signalling. To explore the role of NR3C1 in NIPAs through the regulation of the Wnt signalling pathway, we performed rescue experiments using the GSK3β inhibitor CHIR99021 on NR3C1 knockdown HP75 cells. First, we validated the effect of NR3C1 knockdown on key proteins in the Wnt pathway via Western blot analysis. The results showed that protein expression levels of β‐catenin and p‐GSK3β decreased significantly after NR3C1 knockdown and were partially restored upon the addition of CHIR99021 (Figure 8A). We assessed Wnt pathway activity using the TOP/FOP luciferase reporter assay. The results demonstrated that NR3C1 knockdown significantly reduced Wnt pathway activity, and the addition of CHIR99021 significantly rescued this effect (Figure 8B, *p* < 0.001). Similarly, qPCR analysis showed that NR3C1 knockdown significantly decreased the mRNA expression of the Wnt pathway downstream target genes AXIN2 and c‐Myc, and the addition of CHIR99021 partially restored this expression (Figure 8C, *p* < 0.001). We then assessed cell proliferation capacity. CCK‐8 assay results showed that NR3C1 knockdown significantly inhibited cell proliferation, and the addition of CHIR99021 partially restored proliferative capacity (Figure 8D, *p* < 0.001). EdU incorporation assay results further confirmed these findings, as NR3C1 knockdown significantly reduced the cell proliferation rate, while CHIR99021 addition significantly restored proliferative ability (Figure 8E, *p* < 0.001). Apoptosis was detected via Annexin V‐FITC staining. The results revealed that NR3C1 knockdown significantly increased the apoptosis rate, which was significantly reduced after adding CHIR99021 (Figure 8F, *p* < 0.001). Colony formation assay results indicated that NR3C1 knockdown significantly inhibited the clonogenic ability of pituitary adenoma cells, and this ability was significantly restored upon CHIR99021 addition (Figure 8G, *p* < 0.001). Cell invasive capability was assessed using Transwell migration and invasion assays. The results showed that NR3C1 knockdown significantly inhibited cell migration and invasion abilities, and these capabilities were significantly enhanced after adding CHIR99021 (Figure 8H, *p* < 0.001). These results further confirm the importance of NR3C1 as a potential therapeutic target.

### 3.6 NR3C1 Influences the Growth and Invasiveness of Mouse Subcutaneous Xenograft Tumours of Non‐Functional Invasive Pituitary Adenoma by Regulating the Wnt Signalling Pathway

**FIGURE 9:**
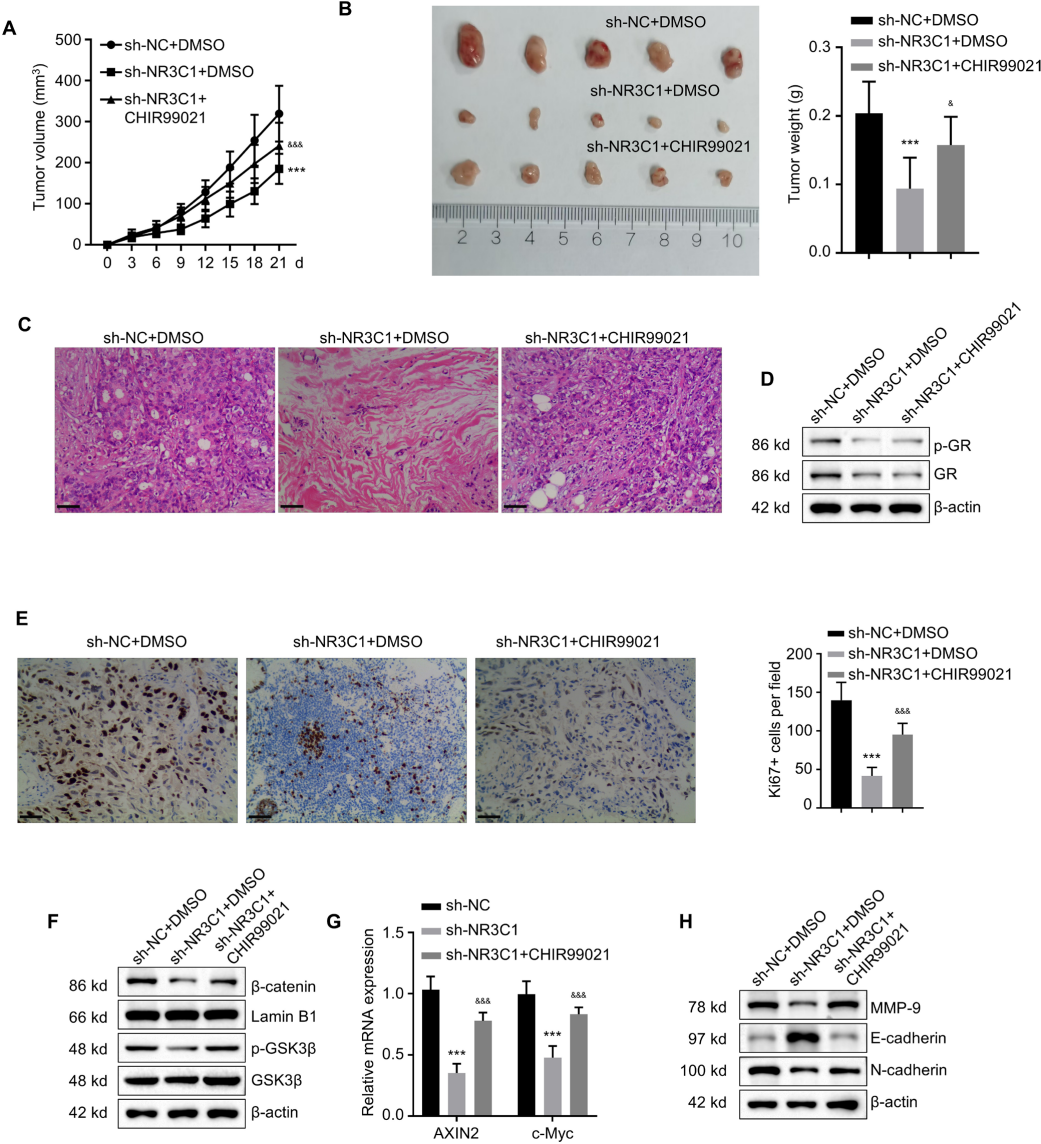
Effect of NR3C1‐mediated Wnt signalling pathway activation on mouse subcutaneous xenograft tumours. (A) Tumour volume measurements showing NR3C1 knockdown significantly inhibited tumour growth, and CHIR99021 significantly restored tumour volume (*p* < 0.001). (B) Tumour weight measurements showing NR3C1 knockdown significantly reduced tumour weight, and CHIR99021 significantly increased tumour weight (*p* < 0.001). (C) H&E staining results showing significantly reduced tumour cell density in the NR3C1 knockdown group, partially restored in the CHIR99021 treatment group. (D) Western blot analysis showing NR3C1 knockdown significantly reduced p‐GR and GR expression levels, partially restored in the CHIR99021 treatment group. (E) Immunohistochemical staining results showing Ki67‐positive cells were significantly reduced in the NR3C1 knockdown group and significantly increased in the CHIR99021 treatment group (*p* < 0.001). (F) Western blot analysis showing expression of β‐catenin and p‐GSK3β was significantly decreased in the NR3C1 knockdown group and significantly restored in the CHIR99021 treatment group. (G) qPCR results showing expression of downstream genes AXIN2 and c‐Myc were significantly decreased after NR3C1 knockdown and significantly restored after CHIR99021 treatment (*p* < 0.001). (H) Western blot analysis showing NR3C1 knockdown significantly reduced MMP‐9 and N‐cadherin expression while increasing E‐cadherin expression; CHIR99021 partially restored these expression levels. **p < 0.01, ***p < 0.001, &, *p* < 0.05, &&&, *p* < 0.001 compared with sh‐NR3C1+DMSO.

To investigate the impact of NR3C1, via regulation of the Wnt signalling pathway, on the growth of mouse subcutaneous xenograft tumours of non‐functional invasive pituitary adenoma, we conducted in vivo experiments. By injecting pituitary adenoma cells (TtT/GF cells) into nude mice and treating them with DMSO, NR3C1 knockdown and the GSK3β inhibitor CHIR99021, we evaluated their effects on tumour growth. Tumour volume measurements showed that NR3C1 knockdown significantly inhibited tumour growth, and the addition of CHIR99021 significantly restored tumour growth (Figure 9A, *p* < 0.001). After extraction and weighing, tumours from the NR3C1 knockdown group showed a significant reduction in weight, while CHIR99021 treatment significantly increased tumour weight (Figure 9B, *p* < 0.001). H&E staining results further validated morphological changes in the tumour tissue (Figure 9C); tumour cell density was significantly lower in the NR3C1 knockdown group and was partially restored in the CHIR99021 treatment group. Western blot analysis further verified the effects of NR3C1 and CHIR99021 treatment on proteins related to the Wnt signalling pathway. NR3C1 knockdown significantly reduced the protein expression levels of p‐GR and GR (Figure 9D), and CHIR99021 treatment partially restored these expression levels. Immunohistochemical staining results showed that the number of Ki67‐positive cells decreased significantly in the NR3C1 knockdown group, while CHIR99021 treatment significantly increased the number of Ki67‐positive cells (Figure 9E, *p* < 0.001), indicating that CHIR99021 could significantly rescue the proliferation inhibition caused by NR3C1 knockdown. Western blot analysis showed that NR3C1 knockdown significantly reduced the expression levels of β‐catenin and p‐GSK3β, and these protein expressions were significantly restored after CHIR99021 treatment (Figure 9F). Concurrently, qPCR results showed that the mRNA expression of the Wnt pathway downstream genes AXIN2 and c‐Myc decreased significantly after NR3C1 knockdown and was partially restored by CHIR99021 treatment (Figure 9G, *p* < 0.001). Furthermore, Western blot analysis revealed that NR3C1 knockdown significantly reduced the expression of MMP‐9 and N‐cadherin, while E‐cadherin expression increased (Figure 9H), indicating a significant impact of NR3C1 on tumour cell invasive behaviour, and CHIR99021 could partially restore the expression of these invasion‐related proteins. These data demonstrate that NR3C1 significantly influences the growth and invasive behaviour of mouse subcutaneous xenograft tumours of non‐functional invasive pituitary adenoma by regulating the Wnt signalling pathway. CHIR99021 treatment could partially rescue the inhibitory effects induced by NR3C1 knockdown, further supporting the role of NR3C1 as a potential therapeutic target.

## 4 Discussion

In clinical medicine, NIPAs are recognised as a common type of brain tumour characterised by significant invasiveness, which directly leads to poor patient prognosis [22]. Currently, the understanding of the invasive mechanisms of NIPAs remains insufficient, particularly at the molecular level, where research is not yet deep, posing significant challenges for related treatment strategies and prognosis assessment. Therefore, in‐depth exploration of the invasive mechanisms of NIPAs, especially the role of NR3C1, is of great importance for improving clinical treatment and patient outcomes. This study focuses on NR3C1 and its regulatory role in the Wnt signalling pathway, investigating its impact on the invasiveness and immune characteristics of NIPAs. Utilising multiple omics methods such as RNA‐seq, mass spectrometry, and WGCNA, this study aims to identify key genes associated with NIPA invasiveness and explore the specific mechanisms by which NR3C1 regulates the Wnt signalling pathway. Through the integrated analysis of multi‐omics data, this study not only reveals the molecular features of NIPAs but also provides foundational data support for their invasion mechanisms, potentially offering new diagnostic and therapeutic targets for clinical practice.

NR3C1, as an important transcription factor, plays a key role in the development and progression of various tumours. Recent studies have shown that NR3C1 not only exhibits significant expression changes in PAs but is also closely related to biological behaviours such as tumour cell proliferation, apoptosis and migration [23]. The correlation between NR3C1 and immune‐related features implies that NR3C1‐driven tumour progression may indirectly shape the tumour immune microenvironment, though the exact mechanism requires further validation. In studies of cellular biological behaviour, knockdown of NR3C1 significantly inhibited the proliferation of pituitary adenoma cells and increased the apoptosis rate. This indicates that NR3C1 plays an important role in maintaining tumour cell survival and proliferation. Further experiments also found that activation of NR3C1 promoted cell migration and invasion capabilities, which are closely related to its role in tumour progression. Previous research found that in an ovarian cancer model, psychological/physiological chronic stress activates NR3C1, thereby accelerating epithelial‐mesenchymal transition (EMT) and enhancing metastatic potential. NR3C1 directly promotes NUPR1 transcription, which then upregulates the key EMT driver SNAI2, forming an NR3C1→NUPR1→SNAI2 cascade axis that systematically drives the EMT programme and distant dissemination [14]. In breast cancer, GR can upregulate cell motility‐related genes by forming a GR‐PRMT5‐HP1γ transcriptional complex, enhancing tumour cell migration ability [24]. In castration‐resistant prostate cancer (CRPC), the sLZIP protein enhances matrix degradation and metastatic capability by antagonising GR's transcriptional repression of MMP‐13 [25]. Furthermore, PlexinB1 signalling activation promotes GR nuclear translocation and enhances the expression of migration‐related genes [26].

The immune microenvironment plays a crucial role in tumour invasiveness and metastasis. NR3C1 (glucocorticoid receptor) also shows an important role in regulating the immune microenvironment. Studies have found that the expression of NR3C1 is closely related to the expression of various immune checkpoint genes (such as PD‐L1 and CTLA‐4), which play key roles in tumour immune escape [27, 28]. High expression of NR3C1 is associated with the infiltration status of immune cells in the tumour microenvironment, especially the infiltration of CD8^+^ T cells, suggesting that NR3C1 may affect tumour invasiveness by regulating the function of immune cells [29, 30]. In a hepatocellular carcinoma model, high NR3C1 expression significantly enhanced tumour invasiveness. In mouse xenograft models, overexpression of mitochondrial GR led to accelerated tumour growth, accompanied by reduced oxidative phosphorylation (OXPHOS) biosynthesis, inhibited pyruvate dehydrogenase (PDH) activity, and a shift in glucose metabolism towards glycolysis. These changes promote energy metabolic reprogramming in tumour cells, thereby increasing malignant behaviour [31]. In malignant adrenocortical tumours, NR3C1 expression is significantly upregulated, and immunohistochemical analysis showed strong positive nuclear staining in 94% of malignant adrenocortical carcinomas (ACC), while benign adenomas (ACA) were mostly negative. This high expression is associated with a malignant tumour phenotype and may serve as a diagnostic marker, suggesting that GR plays a promoting role in tumour progression [32]. In lung squamous cell carcinoma, high NR3C1 expression is significantly associated with an increased risk of recurrence. Spatial transcriptomic analysis revealed that tumours with high NR3C1 expression exhibited therapy‐resistant features, and immunohistochemistry confirmed that higher expression levels predicted an increased risk of recurrence, indicating that the GR pathway plays a key role in promoting tumour heterogeneity and malignant transformation [33].

This study found a significant association between NR3C1 and key proteins of the Wnt signalling pathway, suggesting that NR3C1 may affect tumour cell proliferation and migration by regulating the activity of the Wnt signalling pathway. TOP/FOP luciferase assay results further confirmed the impact of NR3C1 on downstream target genes of the Wnt pathway. In the mouse xenograft model, knockdown of NR3C1 significantly inhibited tumour growth, and the rescue effect of CHIR99021 on NR3C1 knockdown cells further supports the importance of NR3C1 in tumour progression. In various epithelial cells (such as skin, retinal, bronchial epithelial cells) and fibroblasts, GR can rapidly activate the phospholipase C (PLC)/protein kinase C (PKC) cascade signalling [34]. This non‐genomic signalling pathway leads to the inactivation of glycogen synthase kinase‐3β (GSK‐3β), thereby stabilising and activating β‐catenin, the core transcriptional co‐activator of the Wnt signalling pathway [34]. The activation of β‐catenin ultimately induces the expression of its downstream target genes (such as the pro‐oncogene c‐myc), thereby promoting tumour cell proliferation and migration. Research in osteosarcoma cells found that GR can directly bind to β‐catenin to form a complex [35], suggesting a direct physical regulatory capacity of GR over β‐catenin, the core molecule of the Wnt pathway. GR may also indirectly affect Wnt pathway activity by regulating metabolic pathways such as fatty acid oxidation (FAO) [36], while promoting tissue fibrosis, it may also provide a favourable microenvironment for tumour progression [36, 37].

## 5 Limitations

Despite providing new insights into the NR3C1‐Wnt axis in NIPAs, this study has limitations. The relationship between NR3C1 and the tumour immune microenvironment is correlative; although IHC and transcriptomic data suggest predominant expression in tumour cells, low‐level expression in infiltrating immune cells cannot be excluded. Functional experiments assessing direct regulatory effects of NR3C1 on immune cells are lacking, and indirect mechanisms linking NR3C1‐driven invasiveness to immune alterations remain unclear. The multi‐omics analyses were based on a relatively small sample size, necessitating validation in larger, multicenter cohorts. Our in vitro and in vivo models—HP75 and TtT/GF cell lines, and nude mouse xenografts—have inherent constraints, as they do not fully capture the heterogeneity of primary tumours or tumour‐immune interactions. While NR3C1's role in Wnt pathway activation is demonstrated, the direct molecular interactions and potential intermediates remain to be elucidated. Finally, although ROC analyses support its diagnostic potential, prognostic relevance and the efficacy of NR3C1‐targeted therapies require further evaluation. Future studies should expand sample sizes, employ primary cells and immunocompetent models, dissect NR3C1‐Wnt molecular interactions, and assess its prognostic and therapeutic utility.

## 6 Conclusion

This study identified NR3C1 as a key invasion‐related gene in NIPAs through multi‐omics integration and machine learning. NR3C1 is significantly upregulated in tumour tissues and promotes proliferation, migration and invasion in vitro and in vivo. Mechanistically, NR3C1 activates the Wnt signalling pathway, and its downregulation suppresses key Wnt proteins and downstream targets, effects partially rescued by the Wnt agonist CHIR99021. In xenograft models, NR3C1 knockdown markedly inhibited tumour growth. Collectively, these findings suggest that NR3C1 drives invasive progression in NIPAs via Wnt signalling and may serve as a potential diagnostic biomarker and therapeutic target. Future research should validate its prognostic value, clarify interactions with other signalling pathways, and explore targeted inhibitory strategies against NR3C1.

## Author Contributions

Xiaoping Wang: experimentation; Xiaoping Wang, Jinfeng Zhang, Tao Jiang, Zhijun Yang and Yu Zhang: writing the original draft, review, editing and conceptualisation; Yuanxiang Lin, Pinan Liu and Xiaoping Wang: methodology, data curation, conceptualisation and writing the original draft.

## Ethics Statement

The human tissue experimental protocol involved in this study was approved by the Ethics Committee of Beijing Tiantan Hospital affiliated to Capital Medical University (KY2022‐172‐03). All procedures performed in studies involving human participants were in accordance with the ethical standards of the institutional and/or national research committee and with the 1964 Helsinki declaration and its later amendments or comparable ethical standards.

## Consent

All cases provided the informed consent.

## Conflicts of Interest

The authors declare no conflicts of interest.

## Supporting information


**Table S1:** The list of 1736 immune‐related genes from the GeneCards database.

## Data Availability

The datasets generated during and/or analysed during the current study are not publicly available, but are available from the corresponding author on reasonable request.
